# RNA binding protein RBM14 promotes radio-resistance in glioblastoma by regulating DNA repair and cell differentiation

**DOI:** 10.18632/oncotarget.1924

**Published:** 2014-04-27

**Authors:** Ming Yuan, Charles G. Eberhart, Mihoko Kai

**Affiliations:** ^1^ Department of Radiation Oncology, Johns Hopkins University School of Medicine, Baltimore MD 21231 USA; ^2^ Department of Pathology, Johns Hopkins University School of Medicine, Baltimore MD 21231 USA

**Keywords:** GBM, tumor-initiating cell, DNA repair, stem cell maintenance, radio-therapy

## Abstract

Glioblastoma multiforme (GBM) is the most aggressive and lethal type of brain tumor. Standard treatment for GBM patients is surgery followed by radiotherapy and/or chemotherapy, but tumors always recur. Traditional therapies seem to fail because they eliminate only the bulk of the tumors and spare a population of stem-like cells termed tumor-initiating cells. The stem-like state and preferential activation of DNA damage response in the GBM tumor-initiating cells contribute to their radio-resistance and recurrence. The molecular mechanisms underlying this efficient activation of damage response and maintenance of stem-like state remain elusive. Here we show that RBM14 controls DNA repair pathways and also prevents cell differentiation in GBM spheres, causing radio-resistance. Knockdown of RBM14 affects GBM sphere maintenance and sensitizes radio-resistant GBM cells at the cellular level. We demonstrate that RBM14 knockdown blocks GBM regrowth after irradiation *in vivo*. In addition, RBM14 stimulates DNA repair by controlling the DNA-PK-dependent non-homologous end-joining (NHEJ) pathway. These results reveal unexpected functions of the RNA-binding protein RBM14 in control of DNA repair and maintenance of tumor-initiating cells. Targeting the RBM14-dependent pathway may prevent recurrence of tumors and eradicate the deadly disease completely.

## INTRODUCTION

The cancer stem cell model of tumor development and progression states that tumors, like normal adult tissues, contain a subset of cells that both self-renew and give rise to differentiated progeny[1]. These cancer stem cells, also called tumor-initiating cells, functionally resemble tissue–specific stem cells, and are thought to be responsible for failure of radio- and chemotherapies[2].

We performed a human whole genome-wide shRNA screening to identify novel genes that cause radio-resistance in GBM spheres. We selected 51 positive hits (p<0.05) that were found multiple times (different shRNAs for the same genes) in two independent samples from GBM-2 spheres. Among them, 27 were named genes, including BRCA1/2, Rad17, p53, Survivin, and Integrin. We selected one of the candidate genes, RBM14, for further study, because it has been implicated in stem cell maintenance as well as DNA damage response[3, 4] that could be responsible for radio-resistance and recurrence of GBM.

RBM14 (also called CoAA, RRM-containing Coactivator Activator) contains two RRM (RNA recognition motif) domains, binds other coactivators to play an important role in the regulation of transcription and is also involved in alternative splicing[5, 6]. RBM14 regulates transcription-coupled splicing, and its own pre-mRNA transcript is alternatively spliced[7]. RBM14 is highly expressed in embryonic stem cells, and its expression is decreased during differentiation[8]. The RBM14 gene at chromosome 11q13 is amplified in human cancers, including lung and skin cancers[7]. Furthermore, proteomic analyses have identified RBM14 in DNA-damage response and telomere-maintenance networks[3, 4].

## RESULTS AND DISCUSSION

The findings below describe data from two independent RBM14 shRNAs that were tested with at least two independent GBM sphere lines.

**Figure 1:**
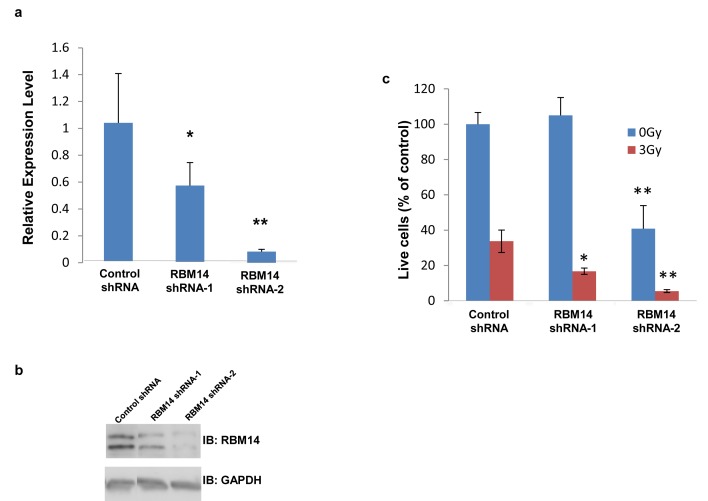
Effects of RBM14 knockdown on clonogenic survival of GBM spheres a, Knockdown of RBM14 expression assessed by qRT-PCR. *p<0.05, **p<0.01. b, Protein levels of RBM14 knockdown cells. c, Viability of shRNA-infected GBM spheres was analyzed with and without irradiation (3 Gy). Numbers of colonies were counted on day 18 after irradiation.

RBM14 knockdown by RBM14-2 (about 90% knockdown) reduced clonogenic survival of GBM spheres, and ionizing radiation (IR) further reduced cell viability. Clonogenic survival of GBM spheres was not affected by shRBM14-1 (about 50% knockdown); however, cells expressing shRBM14-1 showed IR sensitivity (Fig. 1, Supplementary Fig. 1). Similar results were obtained by proliferation assays (Supplementary Fig. 2). RBM14 knockdown did not induce apoptosis judged by caspase-3 activation (data not shown). These results indicate that RBM14 is required for the survival of GBM spheres as well as DNA damage response.

**Figure 2:**
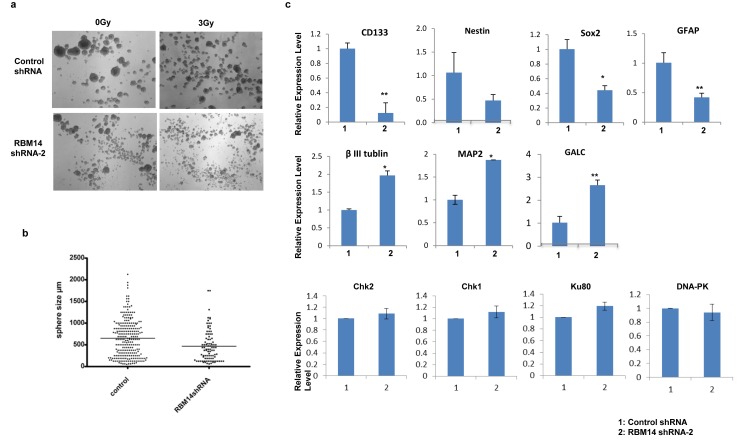
RBM14 knockdown affects GBM sphere size and survival a, Representative images of GBM spheres with control and shRBM14-2 10 days after irradiation. b, Sizes (diameters) of GBM spheres with control shRNA and shRBM14-2. c, Expression levels of stem cell markers, differentiation markers, and DNA damage response genes were determined by qRT-PCR. GAPDH was used as an internal control.

RBM14 is implicated in embryonic stem cell differentiation[8]. Therefore, it is possible that its knockdown affects the differentiation status of GBM stem-like cells. We tested this notion by checking GBM sphere sizes after RBM14 knockdown. shRBM14-2 showed a robust effect. shRBM14-2-infected GBM spheres were not viable after 2 passages in a stem cell medium (Fig. 2a, b). In fact, shRBM14-2 expression induced expression of differentiation markers such as β-III tubulin, MAP2, and GalC, and suppressed expression of stem cell markers such as CD133, and Nestin (Fig. 2c), confirming RBM14's role in the maintenance of GBM stem-like cells. We observed suppression of GFAP expression that is a maker for glioneural progenitor cells and differentiating/differentiated astrocytes in the condition of this experiment (stem cell medium). Consistent with its role in maintaining the stem-like state, RBM14 expression was reduced by serum-induced differentiation (Supplementary Fig. 5), and RBM14 was expressed higher in CD133^+^ cells as compared to CD133^−^ cells (data not shown). RBM14 knockdown by shRBM14-1 did not lead to detectable changes in the sizes of GBM spheres. Therefore, we checked the morphology of CD133^+^ and CD133^−^ cells after RBM14 knockdown by culturing both cells in a medium with serum for 13 days. The CD133^+^ cells maintained the undifferentiated round morphology at this time point, whereas CD133^−^ cells showed more differentiated morphology (flat with elaborated processes), implying that CD133^+^ cells are more resistant to the mitogen-induced differentiation cue. Irradiation did not affect differentiation at 2 Gy. RBM14 knockdown by shRBM14-1 induced apparent morphological changes in CD133^+^ GBM cells in a medium containing serum (Supplementary Fig. 3).

**Figure 3:**
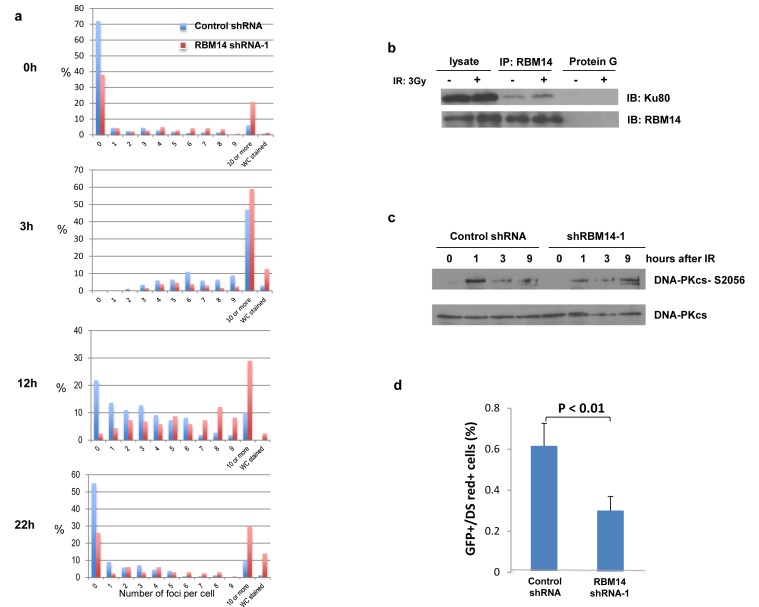
RBM14 knockdown affects repair efficiency a, Numbers of γ-H2AX foci were detected by anti-γ-H2AX antibody. More than 150 cells were analyzed at each time point. b, Ku80 interacts with RBM14, and IR treatment enhances Ku80-RBM14 interaction. RBM14 proteins were immuno-precipitated with an anti-RBM14 antibody, Ku80 protein was detected with an anti-Ku antibody. c, IR (3 Gy)-induced phosphorylation of DNA-PKcs was reduced in shRBM14-1-infected GBM spheres. d, NHEJ frequency was reduced by RBM14 knockdown.

Tumor initiating cells have been implicated in chemo- and radioresistance of GBM[9]. It has been shown that CD133^+^ GBM stem-like cells activate DNA damage response pathways more efficiently than does their CD133^−^ counterpart[9]. Thus we investigated effects of RBM14 in DNA damage response. Knockdown of RBM14 did not affect checkpoint activation in CD133^+^ cells or in total GBM spheres, as judged by Chk2 activation (supplementary Fig. 6). The cell cycle profile was unaffected by shRBM14 (Supplementary Fig. 8). Nonetheless, speed of DSB (double-strand break) repair was significantly affected by RBM14 knockdown (Fig. 3a). The number of γ-H2AX foci induced in GBM spheres decreased in control shRNA-infected cells by 12 hours after IR, whereas shRBM14-infected cells showed significantly more foci at that time point. These results establish that knockdown of RBM14 slows DSB repair in GBM spheres

Recent studies indicate that cancer-initiating cells may take advantage of the mechanisms of DNA repair used by tissue-specific stem cells to mediate resistance to chemo- and radiotherapy[2]. Quiescent tissue specific stem cells utilize the NHEJ mechanism to repair DSBs[10], and RBM14 has been shown to interact with DNA-PKcs and Ku86[6]. Indeed, Ku80 protein was immuno-precipitated by RBM14 pull-down, and RBM14-Ku80 interaction was enhanced by IR treatment (Fig. 3b). Furthermore, we observed reduced DNA-PKcs phosphorylation in GBM spheres after RBM14 knockdown (Fig. 3c). The expression level of DNA-PKcs was not affected by RBM14 knockdown (Fig. 2). Next, we employed an NEHJ assay system[11]. Knockdown of RBM14 reduced NHEJ frequency in GBM spheres (Fig. 3d). More pronounced inhibition of NHEJ was observed with shRBM14-2 (Supplementary Fig. 8). These results indicate that RBM14 is involved in controlling the DNA-PK-dependent NHEJ pathway, and contributes to radiation resistance of GBM spheres.

**Figure 4:**
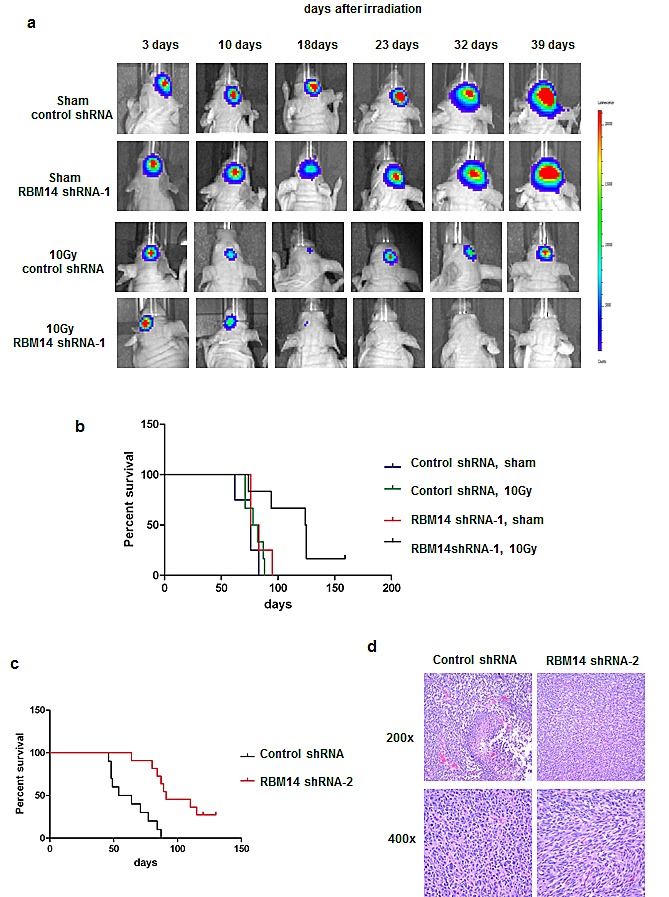
Effects of RBM14 knockdown on GBM tumor growth and mouse survival a, GBM-1 spheres expressing luciferase (10^5^ cells) were xenografted into immune-compromised mice (Stereotaxic coordinate: X(AP)=1.0 mm, Y(ML)=2.0 mm, Z(DV)=-3 mm). After 4 weeks, the whole brain of mice was irradiated or sham-irradiated (10 Gy), and the tumor growth was monitored by imaging. b, Survival of GBM-1 sphere xenografted mice with shRBM14-1 infected cells (Logrank test p<0.05). c, Survival of GBM-1 sphere xenografted mice with shRBM14-2 infected cells (Logrank test p<0.05). d. H&E staining of representative control shRNA and shRBM14-2 xenograft tumors. Control xenografts frequently showed necrotic foci (upper left panel) and highly pleomorphic cytology (lower left panel), while those in which RBM14 had been deplete were comprised of blander spindled cells and lacked necrosis (right panels).

We next tested effects of RBM14 knockdown in GBM tumor formation *in vivo*. GBM spheres were infected with control and shRBM14-1, respectively, and the shRNA-expressing cells were selected and injected intracranially into immunocompromised mice. Both control and shRBM14-1 expressing GBM neurospheres developed large tumor masses (Fig. 4a), consistent with the findings at the cellular level: this knockdown level by shRBM14-1 does not affect the viability of GBM spheres. Irradiation (10 Gy) caused reduction of the tumor size, but, tumors regrew in the control-shRNA GBM sphere grafts and the mice died within 100 days after injection. In contrast, tumors were not detectable in shRBM14-1 sphere grafts after irradiation and the shRBM14-1 mice showed significantly better survival after irradiation (Fig. 4b). The shRBM14-1 GBM sphere grafts developed recurrent tumors much later time points after IR. We observed expression of RBM14 in these tumors (Supplementary Fig. 9, left panel). Therefore, these tumor formations are due to re-expression of RBM14 or to cells that escape RBM14 knockdown. The shRBM14-2 sphere grafts developed tumors much later and survived longer compared to the control shRNA grafts (Fig. 4c). Re-expression of RBM14 was also observed in these tumors (Supplementary Fig. 9, right panel). Histopathological analysis suggested that shRBM14-2 resulted in better differentiated tumors. Anaplasia was more prominent in the controls, while RBM14 knockdown was associated with a blander, spindled phenotype. The control xenografts also frequently contained foci of pseudopalisading necrosis, while these were never observed in shRBM14-2 sphere grafts (Fig. 4d).

In conclusion, we identified a novel protein that regulates both stem-like properties and the NHEJ pathway of GBM spheres. One of the reasons why the stem-like cells repair DNA damage more efficiently might be because the stem-cell maintenance protein also functions in the DNA repair process.

We tested two independent GBM sphere lines, GBM-1 and GBM-2, and observed similar/same results in both lines. We also detected high level of RBM14 expression in at least 6 independent GBM-sphere lines. It is still possible that RBM14-dependent regulations might be only seen in a subset or a subtype of GBMs. GBM-1 spheres have wild-type p53 and IDH1, lacks EGFRvIII, and Rb expression, and PDGFBB is overexpressed (gene amplification status is unknown). GBM-2 spheres show defects in p53-dependent pathway although the protein is expressed, intact Rb expression, and PDGFBB is overexpressed (gene amplification status is unknown). GBM-2 spheres have been classified as type II (similar to adult neural stem cells and the mesenchymal subtype) [12]. However, the subtypes of GBM spheres cannot be easily determined by gene expression profilings, since gene expression patterns can be changed by the culture condition[13]. Therefore, further characterization of a large number of GBM spheres is required to address this question.

Knockdown RBM14 expression slows DNA repair speed by affecting the NHEJ efficiency. Another RNA-splicing factor NONO has also been shown to regulate DNA repair pathways[14-16]. Furthermore, other large-scale screenings of DNA damage response factors have identified RNA processing factors, indicating that RNA processing and DNA repair intersect[17, 18]. It is unknown “how” they intersect, however (i.e. RNA processing might couple with DNA repair pathways, or the two processes might be regulated separately).

Tumor-initiating cells are implicated in the initiation, development, and maintenance of GBMs, and are thought to cause radio/chemo-resistance and recurrence of these tumors[2]. Our results show that the stem cell regulation gene controls DNA damage response, causing radio-resistance of the GBM stem-like population. Targeting of the RBM14-dependent pathway may eradicate GBMs and prevent recurrence by affecting two different pathways - cell differentiation and DNA damage response - that are responsible for the resistance of GBM tumor- initiating cells to treatment.

## Materials and Methods

### Human GBM spheres

GBM-1 was kindly provided by Dr. A.L. Vescovi, University of Milan-Bicocca[19]. GBM-2 was obtained from BioRep s.r.l repository (G179)[20]. GBM-3 was generated from a patient undergoing resection, in accordance with a protocol approved by Johns Hopkins University Institutional Review Board. GBM spheres were cultured in a “stem cell medium” as described in[21] to enrich tumor-initiating cells.

### Human whole-genome-wide shRNA screening

The human whole-genome shRNA library (Decode RNAi Viral Screening Library, Open BioSystems) was infected into GBM2 spheres. The cells were grown in three separate T75 flasks (4.3 million cells each) with a stem cell medium (triplicate). The neurospheres were broken into single cells by accutase (Sigma) and one million viral particles (a multiplicity of 0.3 to achieve 1 integrant per cell) were infected. After 24 hours of infection, viruses were removed and cells were recovered in NBM medium without growth factors (stem cell medium) for 2 days in the presence of puromycin. Then the transduced cells were irradiated, and were cultured in the presence of puromycin. Over a 4-week culture period, samples were collected on day 3 after infection (T=0), and then once each 2 weeks for 4 weeks (T=1 and T=2). shRNA barcodes were PCR-recovered from genomic samples and competitively hybridized to microarrays (Aligent Technology) with the barcode probe sequences. We selected positive hits (p<0.05) that were found multiple times (different shRNAs for the same genes) in both T=1 and T=2 samples.

### RBM14 knockdown

Negative control and RBM14 shRNAs were obtained from OpenBio Systems (Negative controls: RHS4346, RHS4080, shRBM14: V2LHS_178055 (shRBM14-1), TRCN0000072695 (shRBM14-2)). The shRNAs were infected to GBM spheres, and shRNA-expressing cells were selected for puromycin (1 μg/ml) resistance for 3 days.

### Radiation treatment

Cells were irradiated at indicated doses with Gamma Cell 40A (γ-ray). Mouse whole-brain irradiation (10 Gy) was performed using Shepherd Mark 1 (γ-ray).

### Clonogenic assay

Clonogenic survivals of shRNA-infected cells were determined as described[9]. Briefly, GBM spheres were irradiated in a stem cell medium without growth factors for 24 hours, and then cultured in zinc option media (Life Technologies) with 10% FBS. After 18 days, cells were fixed and stained with 0.05% crystal violet in 50% methanol.

### GBM sphere formation assay

GBM spheres were dissociated, sorted for 1 cell/well in 96-well plates, and cultured in a stem cell medium for 28 days. Visible GBM spheres were counted under an inverted microscope (Nikon ECLIPSE TE200-S). About 200 wells were analyzed for control shRNA-infected cells, and about 500 wells were analyzed for shRBM14-infected cells.

### qRT-PCR

qRT-PCR was performed in triplicate by using Absolute blue SYBER green Rox mix (Thermo Scientific). For normalization, GAPDH was used as endogenous control. Primer sequences are shown in supplemental Table 1.

### Cell sorting

GBM spheres were dissociated, and then washed and resuspended in sorting buffer (HBSS containing 0.2% BSA and 2 mM EDTA). Cells were incubated with CD133/2 (293C3)-PE conjugated antibodies (Militenyi Biotec) at 4°C for 20 min. Cell sorting was performed with a MoFlo cell sorter (Dako Cytomation).

### Immunofluorescent Analysis of γ-H2AX foci

shRNA-expressing GBM cells were cultured with adherent condition[20] onto tissue culture slides coated with laminin, and γ-H2AX foci were detected using anti-γ-H2AX antibody (Cell Signaling, 1:200) as described[22].

### Antibodies and Western-blotting

Cell lysis and western blotting were performed as described in [22]. Antibodies: RBM14 (Abcam ab70636, 1:250), Ku80 (Cell signaling #2753, 1:1000), DNAPK-S2056 (Abcam ab18192, 1:500), DNA-PK (Abcam ab1832, 1:200), Chk2-T68 (Cell Signaling #2661, 1:1000), Chk2 (Cell Signaling #3440, 1:1000).

### NHEJ assay

The reporter substrate to analyze NHEJ was obtained from Dr. V Gorbunova, University of Rochester), and the NHEJ assays were performed and the NEEJ frequencies were determined as described in [11].

### Bioluminescence Imaging

The lentiviral transcriptional reporter system that co-expresses GFP and luciferase (SBI) was infected into GBM spheres, and GFP-expressing cells were sorted by FACS. GBM spheres expressing luciferase were injected into immuno-compromised mice. Bioluminescence imaging of xenografts was performed as described[23].

### Evaluation of tumorigenecity by orthotopic injection

Cells were orthotopically transplanted (Stereotaxic coordinate: X(AP)=1.0 mm, Y(ML)=2.0 mm, Z(DV)=-3 mm) after washing and resuspension (10^8^ cells per ml). Survival of mice was analyzed by Kaplan Meier survival analysis with MesCalc software (Mariakerke). Differences were analyzed by the Logrank test.

## SUPPLEMENTARY FIGURES



## References

[1] Ailles LE, Weissman IL (2007). Cancer stem cells in solid tumors. Curr Opin Biotechnol.

[2] Bao S, Wu Q, McLendon RE, Hao Y, Shi Q, Hjelmeland AB, Dewhirst MW, Bigner DD, Rich JN (2006). Glioma stem cells promote radioresistance by preferential activation of the DNA damage response. Nature.

[3] Rajesh C, Gruver AM, Basrur V, Pittman DL (2009). The interaction profile of homologous recombination repair proteins RAD51C, RAD51D and XRCC2 as determined by proteomic analysis. Proteomics.

[4] Giannone RJ, McDonald HW, Hurst GB, Shen RF, Wang Y, Liu Y (2010). The protein network surrounding the human telomere repeat binding factors TRF1, TRF2, and POT1. PLoS One.

[5] Auboeuf D, Honig A, Berget SM, O'Malley BW (2002). Coordinate regulation of transcription and splicing by steroid receptor coregulators. Science.

[6] Iwasaki T, Chin WW, Ko L (2001). Identification and characterization of RRM-containing coactivator activator (CoAA) as TRBP-interacting protein, and its splice variant as a coactivator modulator (CoAM). The Journal of biological chemistry.

[7] Sui Y, Yang Z, Xiong S, Zhang L, Blanchard KL, Peiper SC, Dynan WS, Tuan D, Ko L (2007). Gene amplification and associated loss of 5' regulatory sequences of CoAA in human cancers. Oncogene.

[8] Yang Z, Sui Y, Xiong S, Liour SS, Phillips AC, Ko L (2007). Switched alternative splicing of oncogene CoAA during embryonal carcinoma stem cell differentiation. Nucleic acids research.

[9] Blanpain C, Mohrin M, Sotiropoulou PA, Passegue E (2011). DNA-damage response in tissue-specific and cancer stem cells. Cell Stem Cell.

[10] Mohrin M, Bourke E, Alexander D, Warr MR, Barry-Holson K, Le Beau MM, Morrison CG, Passegue E (2010). Hematopoietic stem cell quiescence promotes error-prone DNA repair and mutagenesis. Cell Stem Cell.

[11] Seluanov A, Mittelman D, Pereira-Smith OM, Wilson JH, Gorbunova V (2004). DNA end joining becomes less efficient and more error-prone during cellular senescence. Proceedings of the National Academy of Sciences of the United States of America.

[12] Lottaz C, Beier D, Meyer K, Kumar P, Hermann A, Schwarz J, Junker M, Oefner PJ, Bogdahn U, Wischhusen J, Spang R, Storch A, Beier CP (2010). Transcriptional profiles of CD133+ and CD133- glioblastoma-derived cancer stem cell lines suggest different cells of origin. Cancer research.

[13] Bhat KP, Balasubramaniyan V, Vaillant B, Ezhilarasan R, Hummelink K, Hollingsworth F, Wani K, Heathcock L, James JD, Goodman LD, Conroy S, Long L, Lelic N, Wang S, Gumin J, Raj D (2013). Mesenchymal differentiation mediated by NF-kappaB promotes radiation resistance in glioblastoma. Cancer Cell.

[14] Straub T, Grue P, Uhse A, Lisby M, Knudsen BR, Tange TO, Westergaard O, Boege F (1998). The RNA-splicing factor PSF/p54 controls DNA-topoisomerase I activity by a direct interaction. The Journal of biological chemistry.

[15] Krietsch J, Caron MC, Gagne JP, Ethier C, Vignard J, Vincent M, Rouleau M, Hendzel MJ, Poirier GG, Masson JY (2012). PARP activation regulates the RNA-binding protein NONO in the DNA damage response to DNA double-strand breaks. Nucleic acids research.

[16] Bladen CL, Udayakumar D, Takeda Y, Dynan WS (2005). Identification of the polypyrimidine tract binding protein-associated splicing factor.p54(nrb) complex as a candidate DNA double-strand break rejoining factor. The Journal of biological chemistry.

[17] Adamson B, Smogorzewska A, Sigoillot FD, King RW, Elledge SJ (2012). A genome-wide homologous recombination screen identifies the RNA-binding protein RBMX as a component of the DNA-damage response. Nature cell biology.

[18] Hurov KE, Cotta-Ramusino C, Elledge SJ (2010). A genetic screen identifies the Triple T complex required for DNA damage signaling and ATM and ATR stability. Genes & development.

[19] Piccirillo SG, Reynolds BA, Zanetti N, Lamorte G, Binda E, Broggi G, Brem H, Olivi A, Dimeco F, Vescovi AL (2006). Bone morphogenetic proteins inhibit the tumorigenic potential of human brain tumour-initiating cells. Nature.

[20] Pollard SM, Yoshikawa K, Clarke ID, Danovi D, Stricker S, Russell R, Bayani J, Head R, Lee M, Bernstein M, Squire JA, Smith A, Dirks P (2009). Glioma stem cell lines expanded in adherent culture have tumor-specific phenotypes and are suitable for chemical and genetic screens. Cell Stem Cell.

[21] Galli R, Binda E, Orfanelli U, Cipelletti B, Gritti A, De Vitis S, Fiocco R, Foroni C, Dimeco F, Vescovi A (2004). Isolation and characterization of tumorigenic, stem-like neural precursors from human glioblastoma. Cancer research.

[22] Shin MH, Yuan M, Zhang H, Margolick JB, Kai M (2012). ATM-dependent phosphorylation of the checkpoint clamp regulates repair pathways and maintains genomic stability. Cell Cycle.

[23] Jamal M, Rath BH, Tsang PS, Camphausen K, Tofilon PJ (2012). The brain microenvironment preferentially enhances the radioresistance of CD133(+) glioblastoma stem-like cells. Neoplasia.

