# Supplementary material for: RNA binding protein RBM14 promotes radio-resistance in glioblastoma by regulating DNA repair and cell differentiation
Source: Oncotarget. 2014 Apr 27;5(9):2820–6. doi: 10.18632/oncotarget.1924 (PMC4058047; doi:10.18632/oncotarget.1924)
Supplement: Supplementary file 1 [file oncotarget-05-2820-s001.pdf]

RNA binding protein RBM14 Promotes Radio-resistance in Glioblastoma by Regulating DNA Repair and Cell Differentiation

Supplementary Material

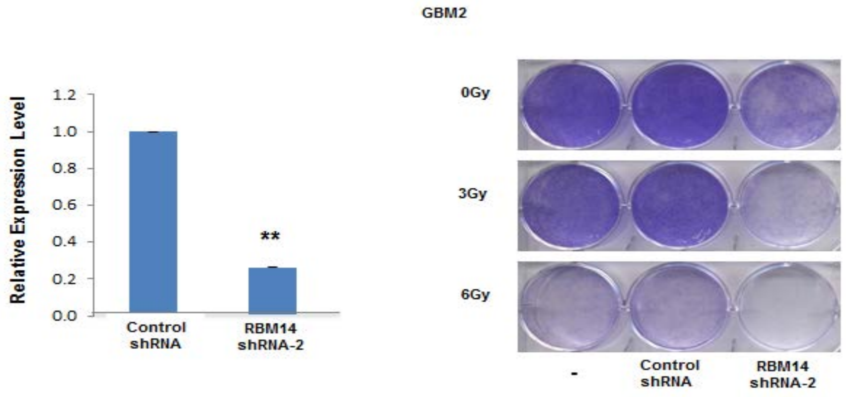

Supplementary Figure 1: Effects of RBM14 knockdown on clonogenic survival of GBM2 spheres. Knockdown levels of RBM14 by shRNAs (left panel). Clonogenic survival of shRBM14-infected cells with indicated doses or without IR (right panel).

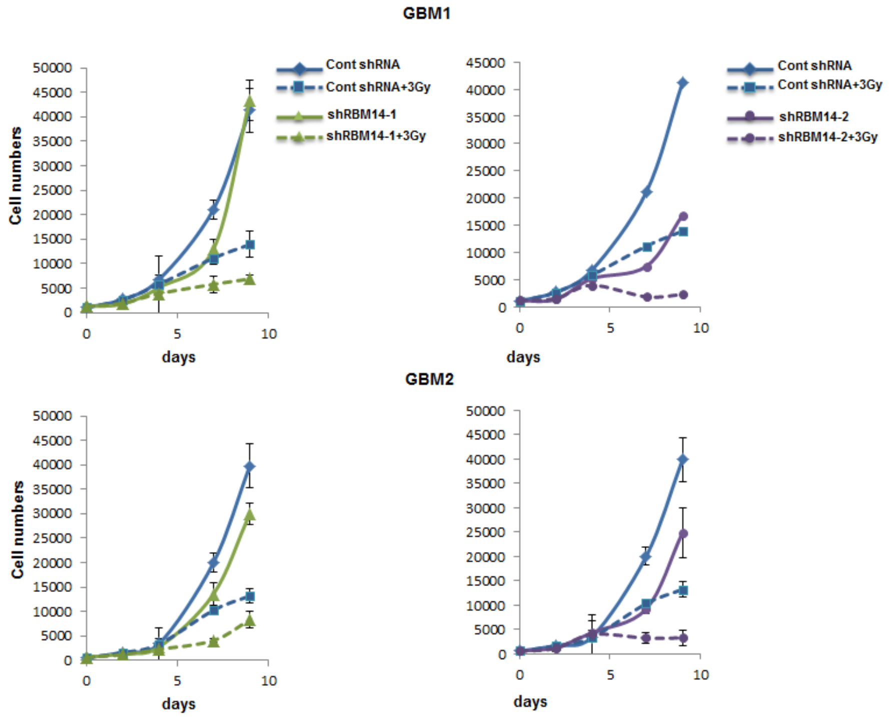

Supplementary Figure 2: Effects of RBM14 knockdown on proliferation and radio-sensitivitiy. shRBM14-2-infected cells (GBM1 & 2) show reduction of growth kinetics. shRBM14-1 does not affect growth, but affects radio-sensitivities of GBM spheres in a stem cell medium.

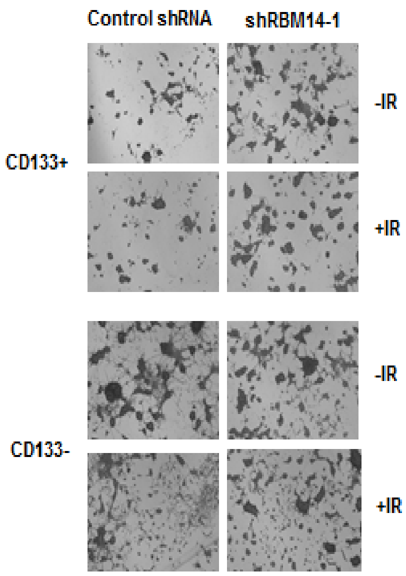

Supplementary Figure 3: shRBM14-1 affects differentiation status of CD133$^+$ cells in serum-containing medium. CD133$^+$ and CD133$^-$ cells were cultured in a medium containing serum for 13 days. IR (2 Gy) did not affect differentiation of shRNA-infected GBM spheres.

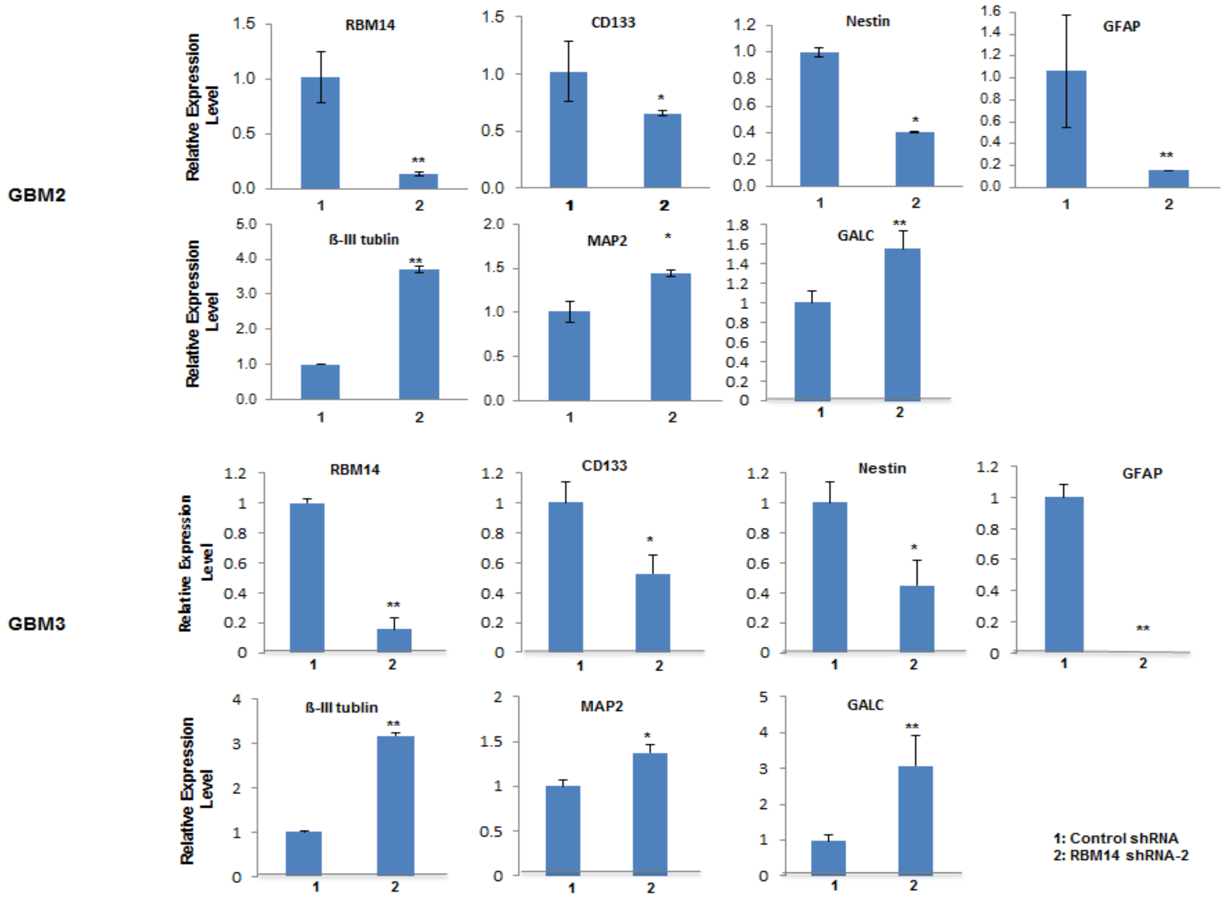

Supplementary Figure 4: shRBM14-2 affects expression levels of stem and differentiation markers. Expression levels of markers in shRNA-infected GBM-2 and GBM-3 were determined by qRT-PCR. GAPDH was used as an internal control.

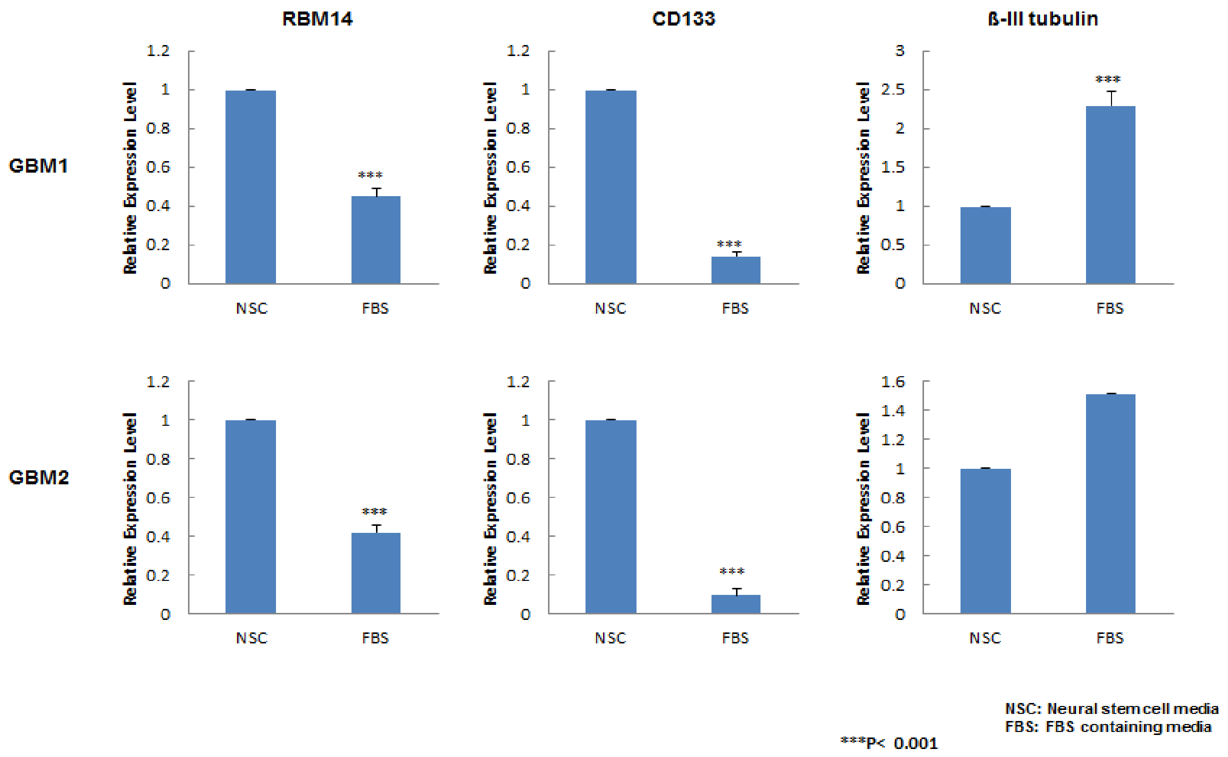

Supplementary Figure 5: RBM14 expression is reduced by serum-induced differentiation. Expression levels of RBM14, CD133 and ß-III tubulin were examined by qRT-PCR in a stem cell medium and a medium containing serum. GAPDH was used as an internal control.

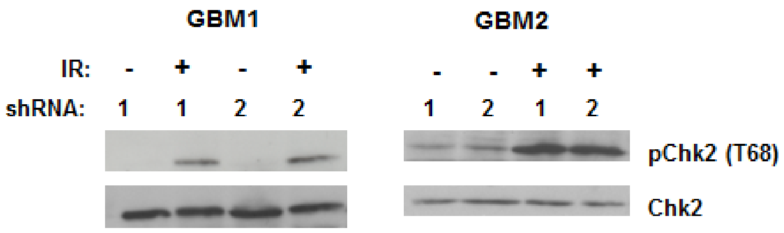

Supplementary Figure 6: RBM14 knockdown does not affect Chk2 phosphorylation after IR. Phosphorylated Chk2-T68 was assessed 1 hour after IR (3 Gy) with GBM-1 and GBM-2 spheres.

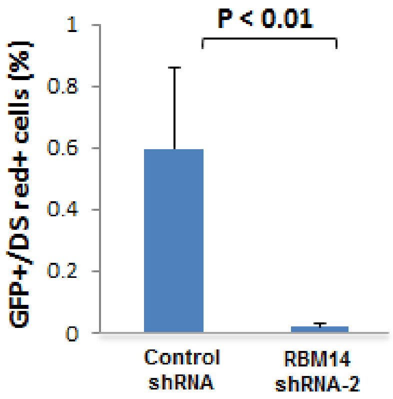

Supplementary Figure 7: RBM14 knockdown affects NHEJ efficiency in GBM spheres. shRBM14-2 reduces the NHEJ frequency in GBM-1 spheres.

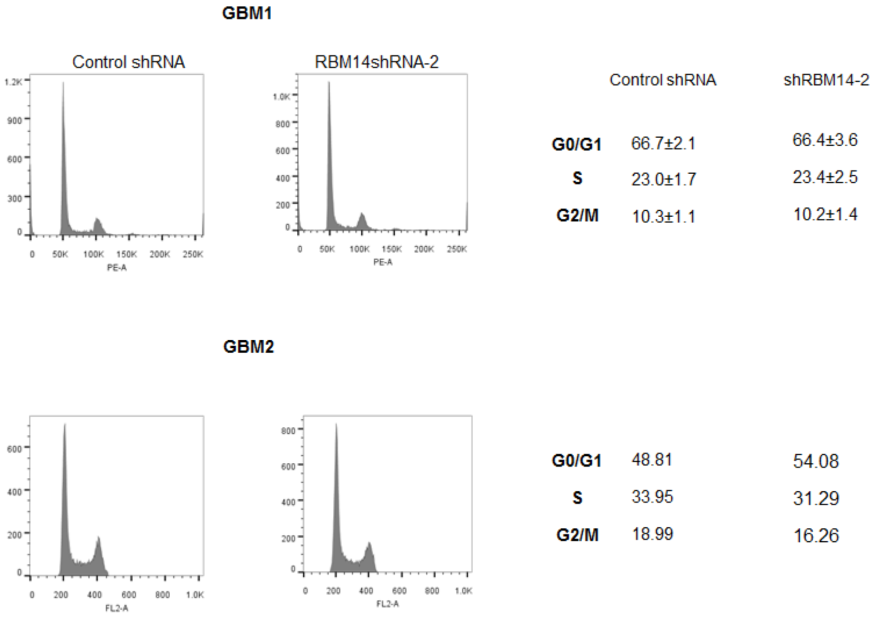

Supplementary Figure 8: RBM14 knockdown does not affect cell cycle profiles of GBM spheres. Cell cycle profiles of shRNA - infected GBM-1 and GBM-2 spheres were analyzed by FACS.

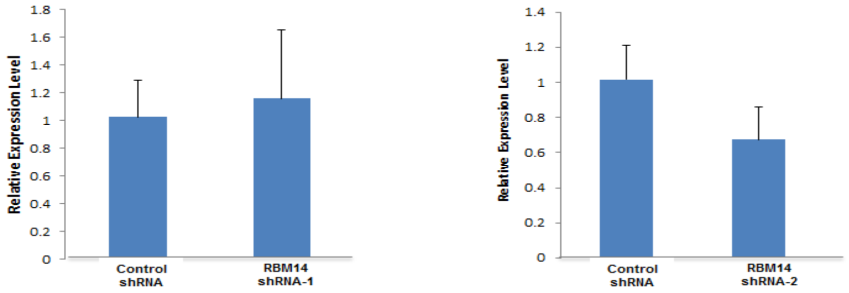

Supplementary Figure 9: Recurrent tumors with RBM14 knockdown xenografts express RBM14. Tumors from RBM14 knockdown xenografts were isolated and RBM14 expression was quantified by qRT-PCR (shRBM14-1; left panel, shRBM14-2; right panel). GAPDH was used as an internal control.
